# A survey on automatic generation of medical imaging reports based on deep learning

**DOI:** 10.1186/s12938-023-01113-y

**Published:** 2023-05-18

**Authors:** Ting Pang, Peigao Li, Lijie Zhao

**Affiliations:** Center of Network and Information, Xinxiang Medical University, Xinxiang, 453000 China

**Keywords:** Medical imaging reports, Automatic generation, Image captioning, Deep learning

## Abstract

Recent advances in deep learning have shown great potential for the automatic generation of medical imaging reports. Deep learning techniques, inspired by image captioning, have made significant progress in the field of diagnostic report generation. This paper provides a comprehensive overview of recent research efforts in deep learning-based medical imaging report generation and proposes future directions in this field. First, we summarize and analyze the data set, architecture, application, and evaluation of deep learning-based medical imaging report generation. Specially, we survey the deep learning architectures used in diagnostic report generation, including hierarchical RNN-based frameworks, attention-based frameworks, and reinforcement learning-based frameworks. In addition, we identify potential challenges and suggest future research directions to support clinical applications and decision-making using medical imaging report generation systems.

## Introduction

**Fig. 1:**
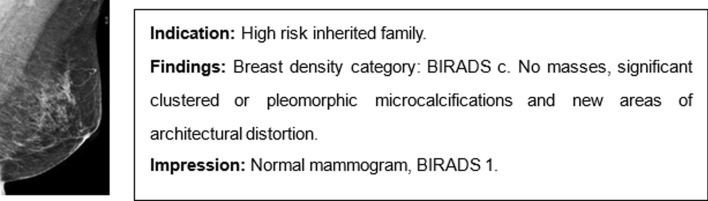
One simple example of mammography report

As we all know, a detailed explanation of medical images such as CT (computed tomography), ultrasound, MRI (magnetic resonance imaging), or pathological imaging must be conducted by professional physicians or pathologists who write a diagnostic report for each patient. An example of such a report can be seen in Fig. 1. Although one report may seem simple, containing only indications, findings and impression, there are many patients with unforeseen abnormal medical images. Therefore, analyzing and depicting textual reports, which require skilled experience, can be a time-consuming and stressful task for professionals. Automatic diagnostic report generation from medical images is an indispensable trend to reduce this workload. In addition, while deep learning, with its advantage of end-to-end processing, has emerged on a large scale in recent medical diagnosis studies, the non-interpretable network and non-standardized evaluation make deep learning like a black box. Teaching machines to automatically write diagnostic reports is a semantic and effective way to support the interpretability of deep learning models [1]. Hence, it is essential to explore the automatic diagnosis of images and the generation of reports to improve the interpretability of deep learning.

**Fig. 2:**
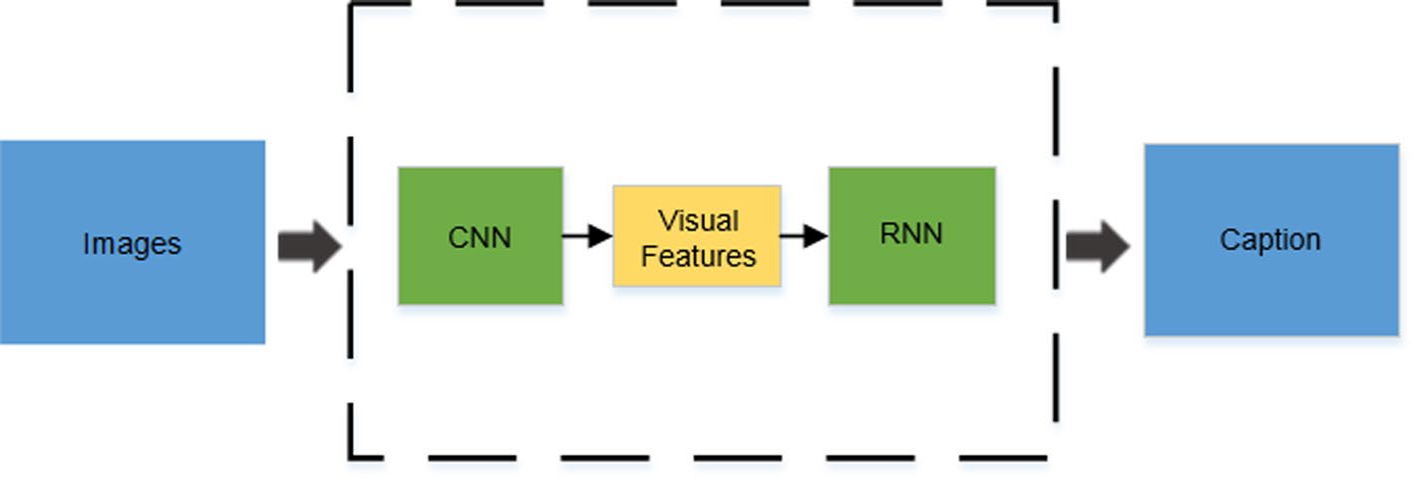
Illustration of the CNN–RNN-based image captioning framework

The automatic generation of diagnostic reports is inspired by image captioning [2], which combines computer vison (CV) and natural language processing (NLP) to provide a comprehensive understanding of medical images. Traditionally, image captioning was achieved through report retrieval [3] and template-based generation [4]. However, these conventional methods are limited in their ability to produce flexible and comprehensive textual descriptions that can be applied to new images. Recent progress in deep learning has led to significant advancements in image captioning. In this research, we focus on medical report generation based on deep learning. Essentially, the paradigm follows a typical encoder–decoder architecture [5–7]. It leverages visual features obtained from Convolution Neural Network (CNN, encoder) to generate descriptions of given images through Recurrent Neural Network (RNN, decoder) [8], as shown in Fig. 2.

However, generating diagnostic reports is a challenging task due to the complexity and diversity of objects in medical images. In practice, the values obtained via the activation function at one suitable layer of the objects recognition CNN are considered as the visual feature vector [9]. Moreover, variations of RNN, such as long–short-term memory (LSTM) [10] and gated recurrent unit (GRU) [11], that contain different controlling gates capable of learning information from a long time ago, are frequently employed in effectively capturing the semantics of image captioning tasks. In addition, more recent works focus on generating long-form text instead of single sentences [12, 13]. Attention mechanisms that focus on salient parts have been widely used in image captioning to provide visual explanations for the rationale of deep learning networks [14–17]. Reinforcement Learning [18] (RL) and Generative Adversarial Networks [19] (GAN) have also been widely proposed in image captioning [20] due to their recent success.

**Fig. 3:**
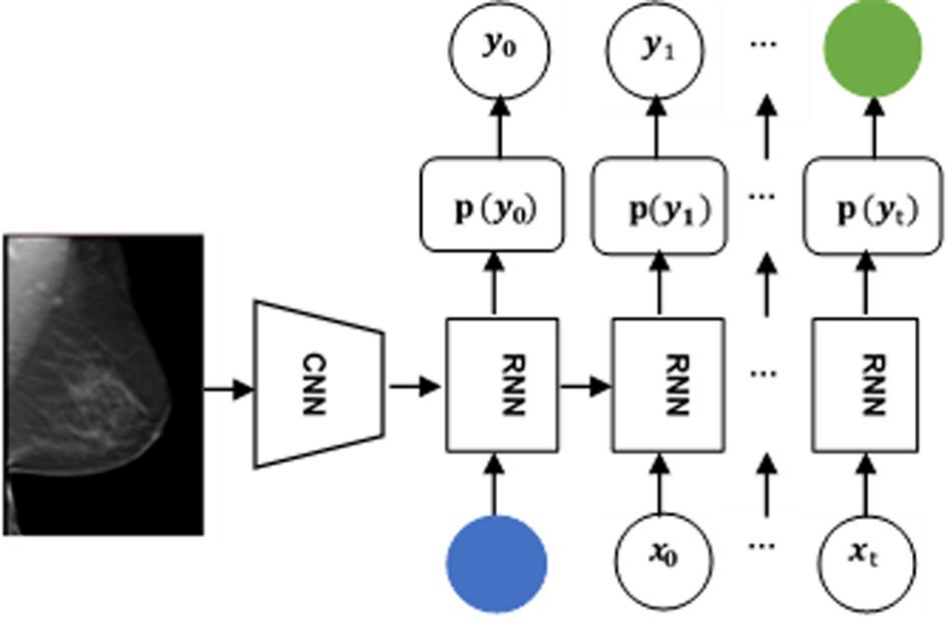
Illustration of the CNN–RNN-based framework for diagnostic report generation. The variable "*t*" represents time, "*x*" denotes the input layer, "*y*" represents the output layer, and "*p*(*y*)" denotes the probability of output

To date, some scholars have explored the automatic generation of medical reports using image captioning methods move forward, see the basic framework in Fig. 3. The first application of deep learning in medical imaging report generation was conducted by Shin et al. [21] in 2016. They developed a CNN–RNN network that effectively predicted only annotated tags (e.g., location, severity and affected organs) from chest X-ray images. They tested both LSTM and GRU and improved the results by considering joint image/text contexts using account using a recurrent neural cascade model. LSTM has been more widely used and studied in the literature, and has achieved state-of-the-art results in many tasks. However, GRU is gaining popularity due to its simpler architecture and faster training time compared to LSTM. Subsequently, in further research on medical image captioning, LSTM will be used as the core framework of RNN.

The primary aim of this manuscript is to present a systematic review of studies on deep learning-based medical imaging reports generation. The survey provides readers with a comprehensive understanding of the field of deep learning in automatic diagnostic reports generation, and to offer clinical treatment management suggestions for medical imaging reports generation exploiting deep learning. The survey also lays the foundation for innovation to increase the richness of this field. To summarize, this work contributes in three ways: (1) it focuses on the clinical value of deep learning-based diagnostic reports generation, providing suggestions for clinical decision making and reducing the workload of radiologists; (2) it organizes and explains the current works in detail, proving that automatic writing diagnostic reports can improve the interpretability of deep learning in medical imaging area; and (3) it provides comprehensive references and identifies new trends for researchers in this field. This paper is the first overview of medical report generation based on deep learning, with a focus on improving interpretability of deep learning and its clinical value.

This paper is structured as follows: in "Overview and analysis" section, we provide a comprehensive summary and analysis of the current state of deep learning applied in medical imaging reports generation, covering aspects, such as data sets, architectures, applications and evaluations based on the retrieved studies. In "Discussion and future" section, we discuss potential challenges and future directions to serve as a reference for further studies in this field. Finally, in "Conclusion" section, we provide brief conclusions.

## Overview and analysis

**Fig. 4:**
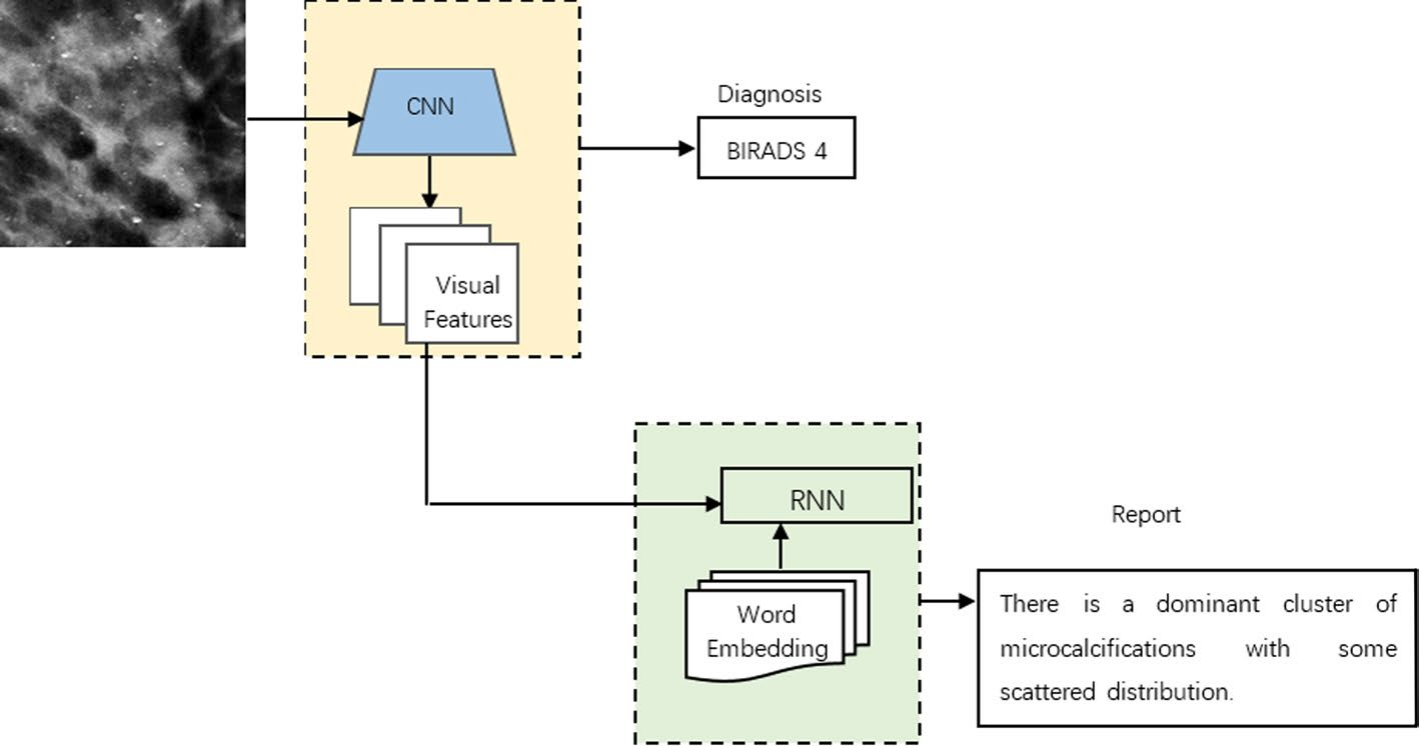
Medical report generation example of the encoder–decoder framework

The encoder–decoder framework, which combines image-text embedding models with multimodal neural language models, was first introduced by [22]. The framework encodes visual data, projecting it into the embedded space composed of RNN hidden states that encode text data by optimizing the pairwise sorting loss. In the embedding space, a structure-content neural language model is used to decode the visual features, based on the feature vectors of context words, to form sentences. An example of the whole framework can be seen in Fig. 4.

Within the framework described above, image captioning is defined as the probability of generating a sentence based on an input image (Eq. 1):

1 $${S}^{*}={}_{S}{}^{argmax}\prod P ({S}_{t} | I, {S}_{0} , . . . , {S}_{t-1} ; \theta )$$

where $I$ is the input image, $\theta$ is the model parameter. A sentence $S$ equals to a sequence of words ${S}_{0} , . . . , {S}_{t-1}$.

Vinyals et al. use a LSTM neural network [8] to model $P ({S}_{t} | I, {S}_{0} , . . . , {S}_{t-1} ; \theta )$ as hidden state ${h}_{t}$, which can be updated as (Eq. 2)

2 $${h}_{t+1}=f ({h}_{t} , {x}_{t} )$$

where ${x}_{t}$ is the input to the LSTM neural network. In the first unit, ${x}_{t}$ is an image feature, while in other units ${x}_{t}$ is a feature of previously predicated context words. The model parameter $\theta$ is obtained by maximizing the likelihood of sentence-image pairs in the training set. Through the training model, the possible output word sequences can be predicted by sampling or beam search.

To generate descriptions closely related to image contents, Jia et al. (2016) extracted semantic information from images and added it to each unit of the LSTM in the process of sentence generation [23]. The original forms of the memory unit and gate of an LSTM unit [24] are defined as (Eqs. 3, 4, 5, 6, 7)

3 $${i}_{l}=\upsigma ({W}_{ix}{x}_{l}+{W}_{im}{m}_{l-1})$$

4 $${f}_{l}=\upsigma ({W}_{fx}{x}_{l}+{W}_{fm}{m}_{l-1})$$

5 $${o}_{l}=\upsigma ({W}_{ox}{x}_{l}+{W}_{om}{m}_{l-1})$$

6 $${c}_{l}={f}_{l}\odot {c}_{l-1}+{i}_{l}\odot h({W}_{cx}{x}_{l}+{W}_{cm}{m}_{l-1})$$

7 $${m}_{l}={o}_{l}\odot {c}_{l}$$

where variables ${i}_{l}$, ${f}_{l}$ and ${o}_{l}$, respectively, denotes input gate, forget gate, output gate of a LSTM cell, ${c}_{l}$ and ${m}_{l}$ denotes the state and hidden state of the memory cell unit, $\upsigma ( \cdot  )$ and $h( \cdot  )$ are non-linear functions, ${x}_{l}$ is the input, $W$ are model parameters, and $\odot$ stands for an elementwise multiplication operation.

Aiming to utilize high-level semantic information for image captioning, Qi et al. (2016) incorporate a set of semantic attributes from the training sentences which are seen as visual concepts into the encoder–decoder framework [25]. In the region-based multi-label classification framework [26], a CNN-based multi-classifier is trained for each attribute. By training the semantic attribute classifiers, the image $I$ can be encoded as a prediction vector ${V}_{att}(I)$ giving the probability of each attribute appearing in the image. Then, a LSTM is deployed as decoder to generate a sentence describing the contents of the image based on the representation. In this case, the image captioning problem can be rephrased as (Eq. 8)

8 $${S}^{*}={}_{S}{}^{argmax}P (S |{V}_{att}(I); \theta )$$

where $I$ is the input image, $\theta$ is the model parameter, $S$ is a sentence.

### Data sets

The automatic generation of medical imaging reports based on deep learning requires a large data set for training. In this section, we introduce frequently used public data sets and some typical private data sets.

The current public data sets have greatly contributed to the development of deep learning for medical imaging report generation. The most commonly used databases consist of images and reports from the United States and Europe, with chest radiographs being the predominant data set. Some examples of these data sets include Indiana University Chest XRay (IU X-Ray) [27], ChestX-ray14 [28], CheXpert [29], MIMIC Chest X-ray (MIMIC-CXR) [30], CX-CHR [31], PadChest [32], as shown in Table 1.

**Table 1:** Common data set of medical imaging report generation

Data set	Description	Image	Report	Link
IU X-Ray	Chest X-ray images of lung diseases	7470	3955	http://openi.nlm.nih.gov/
ChestX-ray14	14 kinds of lung diseases	112,120	–	https://nihcc.app.box.com/v/ChestXray-NIHCC
CheXpert	Chest radiographs of 65,240 patients with lung diseases	224,316	–	https://stanfordmlgroup.github.io/competitions/chexpert/
MIMIC-CXR	227,835 radiographic studies in DICOM format	377,110	227,835	https://physionet.org/content/mimic-cxr/2.0.0/
CX-CHR	Chest X-ray images with Chinese reports of 35,609 patients	45,598	–	–
PadChest	Chest X-ray data set obtained from 67,000 patients	160,000	109,931	https://bimcv.cipf.es/bimcv-projects/padchest/
PEIR Gross	Radiology teaching images	4,000	4000	https://peir.path.uab.edu/library/index.php?/category/106
DDSM	Normal, benign, and malignant mammography studies	2620	–	http://marathon.csee.usf.edu/Mammography/Database.html

The IU X-Ray is a set of chest X-ray images paired with their corresponding diagnostic reports. The data set contains 7470 images (6470:500:500) and 3955 report. Each report consists of the following sections: impression, findings, tags, comparison, and indication. On average, each image is associated with 2.2 tags, 5.7 sentences, and each sentence contains 6.5 words. About 70% of the automatic report generation work are from these public data sets, where IU X-Ray takes up the biggest fraction due to its large numbers and comprehensive annotation.

ChestX-ray14 is provided by the national institute of health (NIH). It comprises 112,120 frontal-view X-ray images of 30,805 (collected from the year of 1992 to 2015) unique patients with the common disease labels, mined from the text radiological reports. The database contains 14 kinds of lung diseases (atelectasis, consolidation, infiltration, pneumothorax, edema, emphysema, fibrosis, effusion, pneumonia, pleural thickening, cardiac hypertrophy, nodules, swelling and hernia).

The CheXpert data set contains 224,316 chest radiographs of 65,240 patients with both frontal and lateral views available. The task is to do automated chest X-ray interpretation, which features uncertainty labels and radiologist-labeled reference standard evaluation sets.

MIMIC-CXR is a large publicly available data set of chest radiographs in DICOM format with free-text radiology reports. The data set contains 377,110 images corresponding to 227,835 radiographic studies performed at the Beth Israel Deaconess Medical Center in Boston. The data set is intended to support a wide body of research in medicine including image understanding, natural language processing, and decision support.

CX-CHR is a proprietary internal data set of chest X-ray images with Chinese reports collected from a professional medical institution for health checking. The data set consists of 35,609 patients and 45,598 images. Each patient has one or multiple chest X-ray images in different views, such as poster anterior and lateral, and a corresponding Chinese report.

PadChest is a labeled large-scale, high resolution chest X-ray data set for the automated exploration of medical images along with their associated reports. This data set includes more than 160,000 images obtained from 67,000 patients that were interpreted and reported by radiologists at Hospital San Juan Hospital (Spain) from 2009 to 2017, covering six different position views and additional information on image acquisition and patient demography. The reports were labeled with 174 different radiographic findings, 19 differential diagnoses and 104 anatomic locations organized as a hierarchical taxonomy and mapped onto standard Unified Medical Language System (UMLS) terminology.

Apart from the chest radiographs, there are some other medical images. Such as PEIR Gross, Digital Database for Screening Mammography (DDSM) [33], etc. PEIR Gross is a collection of over 4,000 curated radiology teaching images, which are created by the University of Alabama for medical education. It contains sentence-level descriptions of 20 different body parts, including the abdomen, adrenal, aorta, breast, chest, head, kidneys, etc. DDSM contains 2620 scanned films of normal, benign, and malignant mammography studies with verified pathology information. It is supported by the University of South Florida and it has been widely used by researchers due to its scale and ground truth validation. Moreover, researchers have trained their deep learning frameworks on several privately owned data sets.

However, private medical imaging data sets are less common. Collecting private medical images can be difficult due to patient confidentiality and data privacy concerns, as well as the laborious effort required for properly indexing, storing, and annotating the images. In addition, image attributes such as cropped image size, format, data source, and number of samples for training and testing can greatly impact the final results [27][28].

### Methods

#### Hierarchical RNN-based framework

**Fig. 5:**
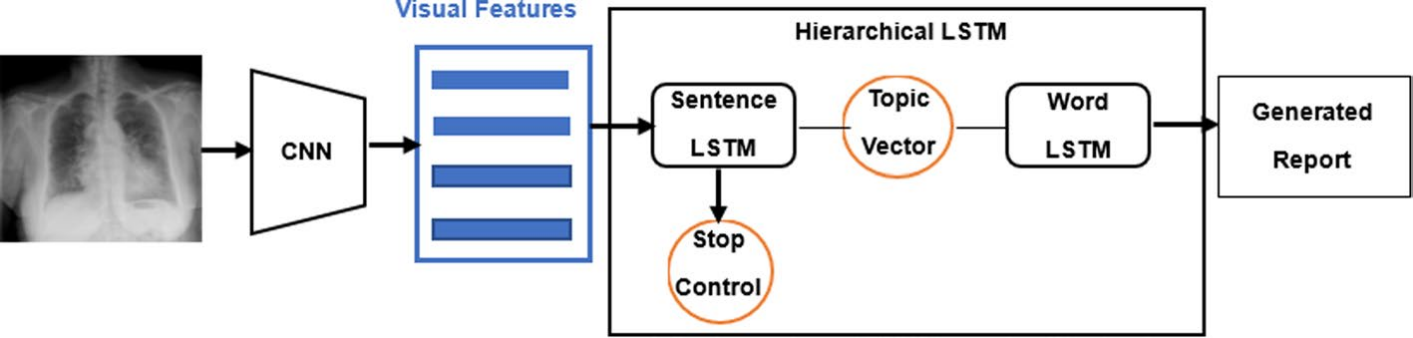
Hierarchical RNN-based framework for medical report generation

As illustrated in Fig. 5, a medical imaging report typically consists of at least one paragraph consisting of several sentences, which can be much longer for abnormal diseases. To address this challenge, Jing et al. proposed a hierarchical LSTM consisting of a sentence LSTM and a word LSTM for generating long chest X-ray reports, inspired by the hierarchical RNN for image captioning proposed by Krause et al. [12]. The single-layer sentence LSTM determines the number of sentences for medical reports using visual features as inputs and generates the topic vector for each sentence, which is then passed to the two-layer word LSTM. The word LSTM generates fine-grained words and descriptions based on the topics for each sentence, which are concatenated to form the final medical report paragraph (see the hierarchical LSTM report generation model in Fig. 5). Harzig et al. also employed hierarchical LSTM to produce diagnostic reports for chest X-ray, and to address data bias, they innovatively proposed dual word LSTMs, including an abnormal word LSTM and a normal word LSTM, which are trained when the label is abnormal and normal [35]. They also set an abnormal sentence predictor to determine whether to use the sentences generated by the dual word LSTM. To address the limited availability of pairs of medical images and reports, Yuan et al. synthesized visual features by taking advantage of multi-view chest X-ray images at the sentence-level LSTM to ensure cross-view consistency [36]. Furthermore, medical concepts based on reports were extracted and merged with respective decoding steps by the word-level LSTM.

#### Attention-based framework

**Fig. 6:**
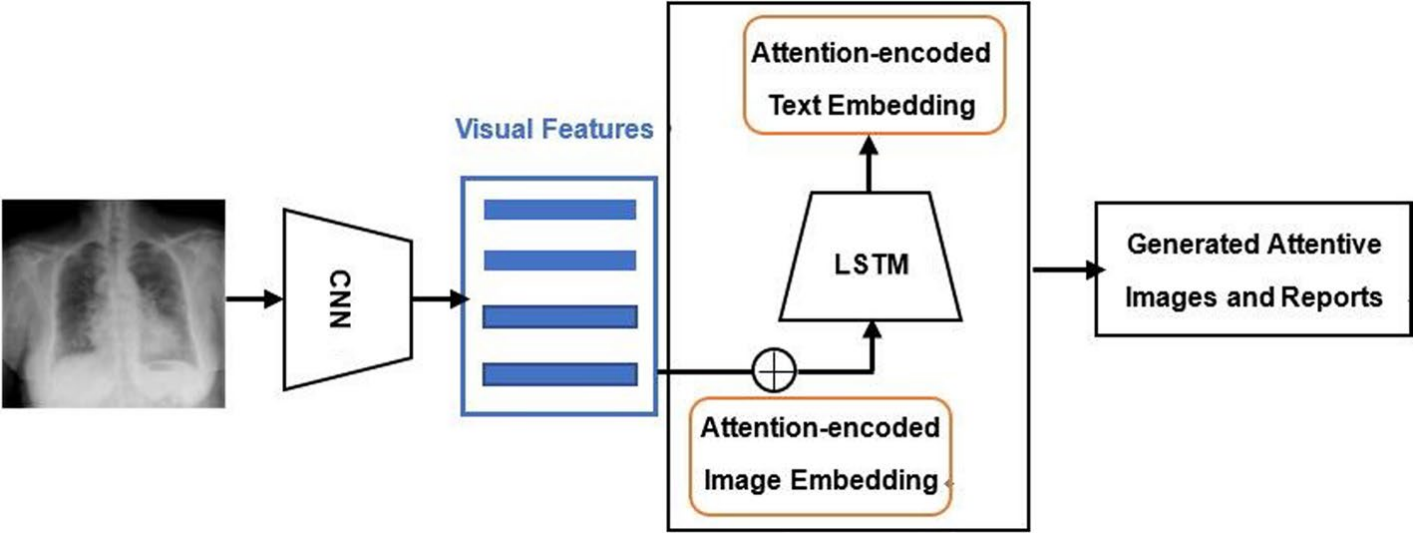
Attention-based framework for medical report generation

Recently, attention-based medical image captioning frameworks have been used to provide meaningful embeddings and improve the interpretability of deep learning processes for report generation (Fig. 6). Zhang et al. built a MDNet for bladder cancer diagnosis that combines an image model and a language model, using an improved attention mechanism to enhance image alignment and generate sharper joint image/report attention maps [37]. Wang et al. proposed TieNet, a multi-level attention mechanism that fuses visual attention and text-based attention into a CNN–RNN model to highlight important report and image representations of chest X-ray patients [38]. Lee et al. designed a justification generator to explain the diagnostic decision of breast masses, utilizing attention to obtain visual pointing maps and an LSTM to generate diagnostic sentences [39]. Li et al. adopted an attentive LSTM that takes either the original chest X-ray image or the cropped abnormal ROI as input and generates the entire report [40].

#### Reinforcement learning-based framework

**Fig. 7:**
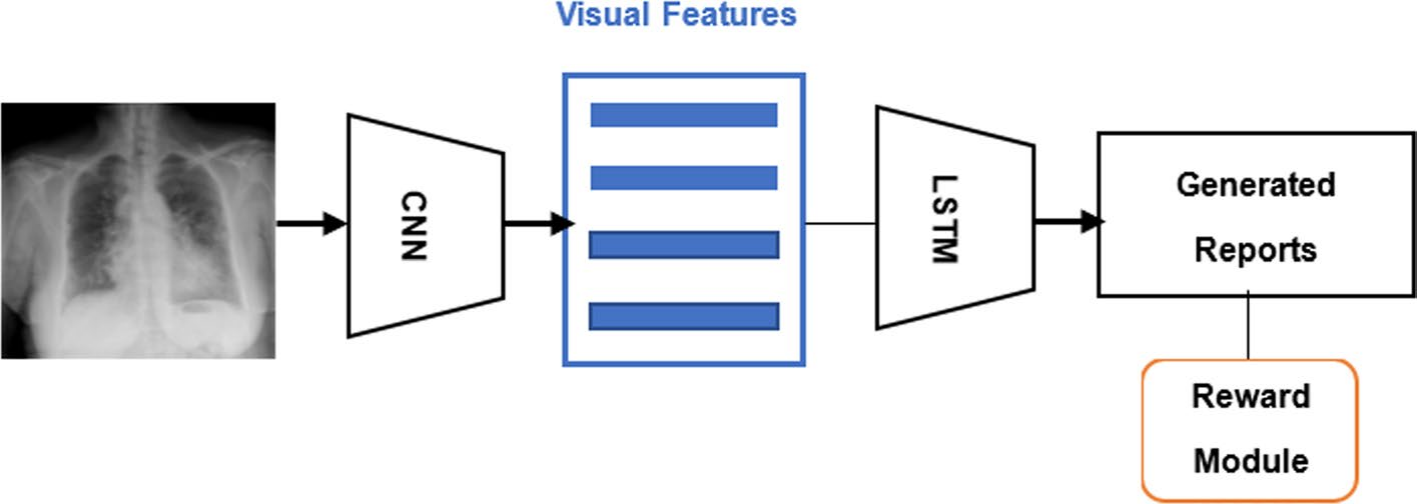
Reinforcement learning-based framework for medical report generation

Motivated by the successful application of reinforcement learning in deep learning, some researchers have attempted to employ RL for optimizing medical imaging report generation, as shown in the basic framework in Fig. 7. RL is formed by agents that learn an optimal policy for better decision-making by receiving rewards from the environment at a given state. Jing et al. proposed a novel Cooperative Multi-Agent System (CMAS) consisting of Planner (PL), Abnormality Writer (AW), and Normality Writer (NW) with one reward module to capture the bias between normality and abnormality for generating more accurate chest X-ray reports [41]. PL determines whether the area has lesions, and AW or NW generates a sentence based on the result given by PL. Similarly, Liu et al. used a final fine-tuned RL containing natural language generation reward and clinically coherent reward to optimize a hierarchical CNN–RNN-based model for clinical accuracy and readability of chest X-ray reports [42].

#### Other-related works

Labeling pairs of medical images and reports is a tedious task for professionals. To address this issue, Han et al. proposed a weakly supervised framework that combines symbolic program synthesis theory and deep learning. This framework uses object-level annotations, without requiring radiologist-level report annotations, to generate unified reports [43]. Similarly, Xue et al. developed a recurrent image captioning model that generates the findings of a medical report sentence by sentence, where each successive sentence is based on multimodal inputs, including the original images and the previous sentence [44]. Zeng et al. introduced a coarse-to-fine ultrasound image captioning ensemble model that helps doctors automatically generate high-quality annotated ultrasound reports [45].

### Applications

The application of automatic generation of medical imaging reports has a wide range of potential benefits beyond assisting diagnosis and lightening workload. For instance, generating accurate and comprehensive reports can improve patient care by providing more informed treatment decisions. In addition, the vast amounts of data generated by medical imaging can be utilized for medical research and advancements in the field. However, efficient and accurate annotation and labeling is required, which can be facilitated by automatic report generation. In summary, the use of deep learning for automatic generation of medical imaging reports has significant potential to greatly benefit the healthcare industry.

#### Assisting diagnosis

Some studies have employed a combination of language models (such as LSTM) and image models (such as CNN) to improve the accuracy of diagnostic conclusions. These models leverage the semantic knowledge of medical images obtained from diagnostic reports to provide an interpretable prediction mechanism. To ensure the reliability of the machine learning system's decisions, it is important to open the black box of deep learning and increase understanding of the reasoning behind the decisions [46]. All the studies reviewed attempt to present semantically and visually interpretable results during the diagnosis process [46–49].

#### Lighten workload

In addition to these modalities and categories of diseases, automatic generation of medical imaging reports has also been explored in other areas such as MRI, CT scans, and PET scans for various diseases such as lung cancer, brain tumors, and cardiovascular diseases. The tedious process of preparing reports can be a significant burden on radiologists and can lead to errors or delays in patient care. By automating this process, radiologists can focus on more complex tasks and improve patient outcomes. Furthermore, the generated reports can provide valuable insights for medical research and contribute to the development of new treatment options.

### Evaluations

BLEU [50], ROUGE [51], METEOR [52] and CIDER [53] are commonly used evaluation metrics for medical image report generation, which are adapted from machine translation and text summarization.

BLEU (Bilingual Evaluation Understudy) measures the similarity between the generated report and the ground truth report by calculating the overlap of word n-grams. BLEU-1 measures the overlap of unigrams (i.e., single words), while BLEU-2, -3, and -4 consider bigrams, trigrams, and quadrigrams, respectively. To account for short generated reports, a penalty is added to the score. BLEU is easy to calculate and interpret, and it has been shown to correlate well with human judgments of text quality. However, BLEU only considers surface-level similarities between the generated and reference texts, and it does not take into account the semantic content or coherence of the generated text.

METEOR (Metric for Evaluation of Translation with Explicit ORdering) extends BLEU-1 by adopting F-score of precision and recall, with a bias towards recall, and utilizing Porter stemmer and WordNet. To account for longer subsequences, it includes a penalty of up to 50% when there are no common n-grams between machine-generated descriptions and references. METEOR takes into account both surface-level and semantic similarities between the generated and reference texts. It also has a built-in mechanism for handling synonyms and paraphrases. Like BLEU, METEOR does not account for the coherence or overall quality of the generated text.

ROUGE-L (Recall-Oriented Understudy for Gisting Evaluation—Longest Common Subsequence) measures the longest common subsequence between the machine-generated description and the reference human description, and calculates its ratio to the reference size (ROUGE-L recall), generated description (ROUGE-L precision), or a combination of the two (ROUGE-L F-measure). ROUGE-L takes into account the semantic content and coherence of the generated text, and it has been shown to correlate well with human judgments of text quality. However, ROUGE-L only considers a single metric, the longest common subsequence, and it may not capture all aspects of text quality.

CIDER (Consensus-based Image Description Evaluation) measures the cosine similarity between n-gram TF–IDF (Term Frequency–Inverse Document Frequency) representations of the generated report and the reference report (words are also stemmed). The calculation is done from single gram to 4 g and the average is returned as the final evaluation score. The rationale behind using TF–IDF is to reward frequent words and penalize common words (such as stop words). CIDER takes into account both surface-level and semantic similarities between the generated and reference texts. It also has been shown to correlate well with human judgments of text quality for image captioning tasks. However, CIDER may not be suitable for tasks other than image captioning, and it is computationally more expensive than other evaluation metrics.

 Automatic generation of medical reports using deep learning is still an emerging area with many challenges. We conducted a search of 31 relevant papers and compiled detailed implementation information in Table 2.

**Table 2:** Studies conducted for medical report generation based on deep learning

References	Data (Images, reports)	Architecture	BLEU-1	BLEU-2	BLEU-3	BLEU-4	MET-EOR	ROU-GE	CIDEr
Shin et al. 2016 [21]	OpenI (7470, 3955)	CNN–RNN	0.972	0.671	0.149	0.028	–	–	–
Zhang et al. 2017 [37]	Bladder Cancer (1000, 5000)	CNN–LSTM–ATT	0.912	0.829	0.75	0.677	0.396	0.701	0.0204
Jing et al.2017 [34]	IU X-Ray (7470, 7470)	CNN–HLSTM–ATT	0.517	0.386	0.306	0.247	0.217	0.447	0.327
Wang et al. 2018 [38]	ChestX-ray14 (−, −)	CNN–LSTM–ATT	0.2860	0.1597	0.1038	0.0736	0.1076	0.2263	–
Xue et al. 2018 [44]	IU X-Ray (7470, 7470)	Recurrent CNN–LSTM–ATT	0.464	0.358	0.270	0.195	0.274	0.366	–
Han et al. 2018 [43]	Lumbar Spinal MRI (253, 253)	Weakly Supervised CNN–LSTM	–	–	–	–	–	–	–
Tian et al. 2018 [54]	CT (−, −)	CNN–LSTM	–	–	–	0.766	–	–	–
Zeng et al. 2018 [45]	Ultrasound Image (−, −)	CNN–LSTM	0.22	0.13	0.09	-	0.10	0.39	0.90
Ma et al. 2018 [55]	Pathology (−, −)	CNN–LSTM	–	–	–	–	–	–	–
Harzig et al. 2019 [35]	IU X-Ray (7470, 3955)	CNN–HLSTM–DualLSTM–ATT	0.373	0.246	0.175	0.126	0.163	0.315	0.359
Yuan et al. 2019 [36]	CheXpert (6248, -)	Muti-view CNN–LSTM–ATT–Medical Concepts	0.529	0.372	0.315	0.255	0.343	0.453	-
Lee et al. 2019 [39]	DDSM FFDM2.0 (605, 605)	CNN–LSTM–ATT	0.4070	0.2296	0.1354	0.0871	-	0.2650	0.1366
Liu et al. 2019 [42]	MIMIC-CXR(327,281, 141,783)	CNN–HLSTM–RL	0.313	0.206	0.146	0.103	0.146	0.306	1.046
Jing et al. 2019 [41]	CX-CHR (−, −)	CMAS–RL	0.428	0.361	0.323	0.290	–	0.504	2.968
Gale et al. 2019 [56]	Frontal Pelvic X-rays (50,363, -)	CNN–LSTM–ATT	0.919	0.838	0.761	0.677	–	–	–
Hasan et al. 2019 [57]	Biomedical Images(164,614, -)	CNN–LSTM	0.3211	–	–	–	–	–	–
Sun et al. 2019 [58]	INbreast (−, −)	CNN–LSTM	–	–	–	–	–	–	–
Xie et al. 2019 [59]	–	CNN–LSTM–ATT	–	–	–	–	–	–	–
Li et al. 2019 [40]	IU X-Ray (7470, 7470)	CNN–LSTM–ATT	0.419	0.280	0.201	0.150	-	0.371	0.553
Yin et al. 2020 [60]	Two image-paragraph pair data sets	Hierarchical RNN	–	–	–	–	–	–	–
Pino et al. 2020 [61]	IU X-Ray (7470, 7470)	CNN–LSTM–ATT	0.361	0.226	0.152	0.106	-	0.314	0.187
Zeng et al. 2020 [62]	Ultrasound image	CNN–LSTM	–	–	–	–	–	–	–
Xu et al. 2020 [63]	IU X-Ray (7470, 7470) and MIMIC-CXR	Reinforce CNN–LSTM	0.412	0.279	0.206	0.157	0.179	0.342	0.411
Singh et al. 2021 [64]	IU X-Ray (−, −)	CNN–LSTM-	23.07	11.86	7.05	4.75	11.11	23.15	19.78
Yang et al. 2021) [65]	Ultrasound image	Adaptive Multimo-dal ATT	–	–	–	–	–	–	–
Najdenkoska et al. 2021 [66]	IU X-Ray (7470, 7470) and MIMIC-CXR	CNN–LSTM–ATT	–	–	–	–	–	–	–
Oa et al. 2021 [67]	IU X-Ray (7470, 7470)	Condition GPT2	0.387	0.245	0.166	0.111	0.164	0.289	0.257
Liu et al. 2021 [68]	COVID-19 cases (1104, 368)	Medical visual language BERT	–	–	–	–	–	–	–
Han et al. 2021 [69]	spinal image data set	Neural-symbolic learning (NSL) framework	–	–	–	–	–	–	–
Wu et al. 2022 [70]	skin pathological image data set (1147, 1147)	CNN–LSTM–ATT	–	–	–	–	–	–	–
Chang et al. 2022 [71]	lung CT scans (458, 458)		–	–	–	–	–	–	–

*ATT* Attention

## Discussion and future

Despite the significant progress made in medical imaging report generation based on deep learning, this section aims to highlight the unresolved issues and present future research directions in this area for further development.

### Balanced data set

Deep learning has shown great potential in big data analytics, but in the field of medical imaging report generation, there are still many challenges to be addressed. One major issue is the imbalanced nature of available data sets. There is a lack of public databases that include a variety of image modalities, such as pathology, ultrasound, and magnetic resonance imaging (MRI). In addition, private data sets are often arbitrary in terms of number, size, and format, which makes it difficult to compare results across studies. Another challenge for both private and public data set is the annotation of images, as clinical radiologists may not always be available due to the labor-intensive and time-consuming nature of the task. The use of imbalanced data sets for training neural networks can lead to biased diagnostic report generation. To address these challenges, we need to establish public databases with a variety of image modalities, as well as develop private data sets to address the limitations of medical images and complex annotations. Private data sets can be useful for clinical practice, such as combining different imaging modalities and diagnostic reports from various sources.

### Clinical application

Clinical decision-making is critical in patient management and care, and errors in medical imaging reporting can lead to serious consequences. Therefore, improving the accuracy of medical reports is crucial. While deep learning has shown great potential in this field, there is still a significant research gap in the domain of diagnostic report generation. Many studies focus on improving the final performance, but we should also pay attention to the deep features obtained by deep learning and consider the unique characteristics of different diseases for accurate report generation. By doing so, we can enhance the practical application value of deep learning in clinical decision-making.

### Unified evaluation

In many studies, the technical details of experiments were not described in sufficient detail. The selection of measurement indicators and baseline methods was often arbitrary, resulting in a lack of standardization in the evaluation process. Most researchers focused on metrics, such as BLEU, ROUGE, METEOR, and CIDER, which are commonly used in natural image evaluation but may not be appropriate for medical imaging reports. To improve the evaluation process, it is necessary to design more specific metrics in the medical domain to better evaluate the accuracy and interpretability of the generated reports.

### Interdisciplinary background

The progress in deep learning for medical imaging report generation is hindered by the lack of collaboration between experts from different fields. Many medical professionals lack the technical expertise to design and code deep learning models, while engineering and computer science specialists may not have sufficient knowledge of medical imaging and complex clinical applications. Better communication and a closer working relationship between these fields are essential to advance deep learning for clinically useful applications in medical imaging report generation.

## Conclusion

Automatic generation of diagnostic reports from medical images can significantly reduce the workload of report writing. In addition, using semantic information to express visual features can improve the interpretability of deep learning-based models. This paper presents a survey of recent studies on deep learning-based medical imaging report generation, organized into four sections: data set, architecture, application, and evaluation. The focus is on frameworks, such as the hierarchical RNN-based framework, attention-based framework, reinforcement learning-based framework, and related works. The paper also discusses potential challenges and future directions for further studies in this area. With the analyzed potential directions for deep learning-based report generation, there are vast opportunities for developments in research and clinical applications. To gain a more specific understanding of the automatic diagnostic report generation procedure, we plan to conduct further studies on private data sets. Specifically, we aim to establish a radiomics-reporting network to improve the interpretability of deep learning and propose text attention to enhance the readability of medical reports.

## Data Availability

Not applicable.
